# Female Advantage in Automatic Change Detection of Facial Expressions During a Happy-Neutral Context: An ERP Study

**DOI:** 10.3389/fnhum.2018.00146

**Published:** 2018-04-19

**Authors:** Qi Li, Shiyu Zhou, Ya Zheng, Xun Liu

**Affiliations:** ^1^CAS Key Laboratory of Behavioral Science, Institute of Psychology, Chinese Academy of Sciences, Beijing, China; ^2^Department of Psychology, University of Chinese Academy of Sciences, Beijing, China; ^3^Department of Psychology, Dalian Medical University, Dalian, China

**Keywords:** sex difference, automatic change detection, facial expression, visual mismatch negativity, pre-attentive processing

## Abstract

Sex differences in conscious emotional processing represent a well-known phenomenon. The present event-related potential (ERP) study examined sex differences in the automatic change detection of facial expressions, as indexed by the visual mismatch negativity (vMMN). As paid volunteers, 19 females and 19 males were presented peripherally with a passive emotional oddball sequence in a happy-neutral context and a fearful-neutral context while they performed a visual detection task in the center of the visual field. Both females and males showed comparable accuracy rates and reaction times in the primary detection task. Females relative to males showed a larger P1 for all facial expressions, as well as a more negative N170 and a less positive P2 for deviants vs. standards. During the early stage (100–200 ms), females displayed more negative vMMN responses to both happy and neutral faces than males over the occipito-temporal and fronto-central regions. During the late stage (250–350 ms), females relative to males exhibited more negative vMMN responses to both happy and neutral faces over the fronto-central and right occipito-temporal regions, but only more negative vMMN responses to happy faces over the left occipito-temporal region. In contrast, no sex differences were found for vMMN responses in the fearful-neutral context. These findings indicated a female advantage dynamically in the automatic neural processing of facial expressions during a happy-neutral context.

## Introduction

Sex differences in emotional processing constitute one of well-known sex stereotypes (Grossman and Wood, 1993; Timmers et al., 2003). For example, females relative to males are more emotionally perceptive, more reactive to emotional stimuli, experience emotions with greater intensity, but are less efficient in emotion regulation (for a review, see Whittle et al., 2011). Sex differences in various aspects of emotional processing are associated with the prevalence of various emotional disorders (Gater et al., 1998; Bao and Swaab, 2010). It is vital to understand the sex difference in brain functions associated with emotional processing (Cahill, 2006; Grabowska, 2017).

Facial expression is an important tool for conveying social-emotional information, and rapid perception and interpretation of facial expression are critical for survival. The perceptual processing of facial expression has been indexed by several event-related potential (ERP) components. The first ERP component is the P1, peaking at approximately 100 ms post stimulus onset at occipital sites. Despite inconsistencies, the effect of emotional facial expression begins as early as the P1, as reflected by larger P1 amplitudes for fearful relative to neutral or happy facial expression (for a review, see Vuilleumier and Pourtois, 2007). Following the P1 is a face-sensitive component called the N170, which is recorded about 130–200 ms post stimulus onset with an occipito-temporal distribution (Bentin et al., 1996). The N170 is enhanced for emotional relative to neutral facial expressions, including anger, fear and happy faces, and this modulation appears to be enhanced during emotion-irrelevant compared to emotion-relevant tasks (for a recent review, see Hinojosa et al., 2015). In addition, another component called the early posterior negativity (EPN) is also sensitive to emotional facial expressions. The EPN is a *relative* negativity with a posterior distribution occurring between 200–300 ms following stimulus onset and appears to reflect early automatic attention capture (Schupp et al., 2003). Recent research has demonstrated that the EPN is more negative for emotional compared to neutral faces (Marinkovic and Halgren, 1998; Rellecke et al., 2011; Itier and Neath-Tavares, 2017; Langeslag and van Strien, 2018).

In electrophysiological studies, several previous studies have demonstrated sex differences in neural responses to emotional facial expressions. For example, using an emotional oddball task, Campanella et al. (2004) found that N2b latency was delayed for happy faces compared to fearful faces in males but not in females. Another study found an enhanced P1 in response to fearful faces over the right hemisphere for female relative to male schizophrenia individuals (Lee et al., 2010). These studies indicate that sex differences in neural responses to emotional facial expressions can be reflected in the early processing stage. However, whereas almost previous studies have focused on the conscious processing of facial expressions, few studies, if any, paid attention to sex differences in the unconscious processing of facial expressions (Donges et al., 2012; Lee et al., 2017). Using a subliminal affective priming paradigm, a previous study found that females relative to males were more perceptive and responsive to happy, instead of sad, facial emotion despite the lack of conscious awareness (Donges et al., 2012). Recently, an ERP study employing a visual backward masking paradigm found that females exhibited larger P1 responses to subthreshold fearful faces than males (Lee et al., 2017). Here, we focus on one specific aspect of the unconscious processing of facial expression: the automatic change detection of facial expressions. We investigate whether females differed from males when facial expressions appeared outside of the focus of visual attention.

The automatic change detection of facial expression is associated with an ERP component called visual mismatch negativity (vMMN). As a counterpart of the auditory MNN (Näätänen et al., 2007), the vMMN is a negative-going wave with a posterior distribution that is maximal between 200–400 ms after stimulus onset (Czigler, 2007). This component is typically elicited by infrequency (deviant) stimuli embedded in a stream of frequency (standard) stimuli with differences in visual features, while participants are performing a primary task unrelated to the oddball task in order to draw their attention. Recent theories propose that the vMMN reflects a prediction error signal, i.e., the difference between a sensory input and the prediction generated by the representation of the repeated standard stimuli in transient memory (Kimura, 2012; Stefanics et al., 2014). This prediction error account has been supported not only by low-level visual features, such as color, orientation, movement, contrast and spatial frequency, but also by high-level visual properties such as facial expressions (for reviews, see Czigler, 2007; Kimura, 2012; Stefanics et al., 2014). Previous research has demonstrated that expression-related vMMN can be observed in multiple time windows (100–400 ms) over bilateral posterior occipito-temporal areas (Zhao and Li, 2006; Astikainen and Hietanen, 2009; Chang et al., 2010; Kovarski et al., 2017), together with frontal areas (Kimura et al., 2012; Stefanics et al., 2012; Liu et al., 2016). According to the prediction error model, the system that produces the expression-related vMMN automatically registers regularities in the emotional expression of unattended faces appearing outside of the focus of attention and then uses them as predictive memory representations, whereby sudden changes in emotional expressions, that is, the violation of these predictive memory representations, would orient attention to such changes for behavioral adaptation (Kimura et al., 2012; Stefanics et al., 2012).

To the best of our knowledge, only one study has investigated sex differences in the automatic change detection of facial expressions (Xu et al., 2013). Adopting schematic emotional faces, Xu et al. (2013) used an oddball task unrelated to participants’ primary task (a visual detection task) and reported that females elicited a larger vMMN for sad faces than for happy faces during the early time window (120–230 ms) over the right hemisphere but not the left hemisphere. By contrast, males failed to exhibit this emotional modulation of the vMMN over both hemispheres. However, there were two limitations in that study. First, facial expressions in that study were manipulated by the direction of the mouth of schematic faces, thus preventing the conclusion of the sex differences in the automatic change detection of facial expressions from generalization. More importantly, although neutral faces were included in that study, vMMN responses to neutral facial expression were not analyzed. It thus remains unclear whether the observed sex differences were associated with emotional facial expressions specifically or facial expressions generally.

Here, this study aimed to address sex differences in the automatic change detection of facial expressions. We compared females’ and males’ vMMN responses to unattended rare (deviants) facial expressions delivered in a stream of frequent (standards) facial expressions (a passive emotional oddball sequence) in the visual periphery while participants were performing a primary change detection task in the center of the visual field. The primary task was employed to draw participants’ attention and was independent of the passive oddball sequence. The oddball sequence included a happy-neutral context during which happy and neutral faces were used as deviants and standards in different blocks, and a fearful-neutral context during which fearful and neutral faces were used as deviants and standards in different blocks. In contrast to Xu et al. (2013) who used schematic sad faces in their study, we employed fearful faces here since they are more representative in previous emotional vMMN studies (for a recent review, see Kovarski et al., 2017). Similar to a previous study (Stefanics et al., 2012), each stimulus screen consisted of four faces of different identity but displaying the same emotion, which were shuffled randomly around four locations in the visual periphery. This protocol allows for subtle control for low-level visual features and only high-level features, that is, the common emotional valence (happy, sad and neutral) across the four faces, can be extracted to establish and maintain predictive memory representations.

Based on previous research (Xu et al., 2013), we hypothesized that females relative to males would be more sensitive to automatic changes in emotional facial expressions, as reflected by enhanced vMMN responses. Given previous evidence of cerebral lateralization for positive and negative emotions (Prete et al., 2015a,b), sex differences in the emotional vMMN responses would possibly show a hemispheric asymmetry. No predictions were made for sex differences in neutral vMMN responses because of the lack of previous findings.

## Materials and Methods

### Participants

As paid volunteers, 19 females (*M* = 33.32 years, *SD* = 7.06) and 19 males (*M* = 31.57 years, *SD* = 7.76) participated in the experiment. All participants were right-handed as determined by self-report. All had normal or corrected-to-normal vision and were free from psychological or neurological disorders. This study was carried out in accordance with the recommendations of the Dalian Medical University Institutional Review Board with written informed consent from all subjects. All subjects gave written informed consent in accordance with the Declaration of Helsinki. The protocol was approved by “the Dalian Medical University Institutional Review Board”.

### Materials and Procedure

**Figure 1:**
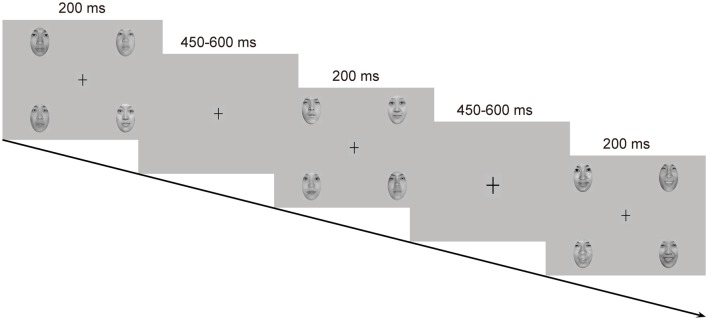
Schematic representation of the passive emotional oddball sequence and the cross-change detection task.

Emotional stimuli were images of facial expressions from 18 Chinese models (9 females and 9 males) in three expressions: happy, neutral and fearful, which were taken from the native Chinese Facial Affective Picture System (CFAPS; Gong et al., 2011). Each stimulus screen consisted of four images of faces (2 females and 2 males) expressing the same emotion (Figure 1). The four faces were selected from the 18 models randomly with the restriction that the face of the same individual was not presented on the next stimulus screen. The four faces were presented in the upper-left, upper-right, lower-left and lower-right part of the stimulus screen, each viewed from a distance of 0.5 m subtending a visual angle of approximately 5.73 × 8.02°. The distance of the center of each face picture from the center of the screen was 7.67° visual angle horizontally and 4.58° visual angle vertically. All faces, cropped into the shape of an ellipse, were presented with only interior characteristics being retained and were similar to one another in size, background, brightness, spatial frequency and contrast grade. Normative valence (1 = negative and 9 = positive) and arousal (1 = low intensity and 9 = high intensity) ratings from the CFAPS were assessed with separate one-way analysis of variance (ANOVA) with emotion (happy, neutral and fearful) as a within-subjects factor. For the valence ratings, there was a significant main effect, *F*_(2,34)_ = 121.77, *p* < 0.000001, $\eta_{\text{p}}^{2}$ = 0.88. *Post hoc* comparisons revealed that the valence-rating scores decreased as a gradient from happy (*M* = 5.69, *SD* = 0.88) to neutral (*M* = 4.15, *SD* = 0.51), and to fearful (*M* = 2.84, *SD* = 0.37) faces (*p*s < 0.0001). Similarly, there was a significant main effect for the arousal ratings, *F*_(2,34)_ = 9.14, *p* = 0.002, $\eta_{\text{p}}^{2}$ = 0.35. *Post hoc* comparisons indicated that the arousal-rating scores were higher for both happy (*M* = 5.56, *SD* = 0.26) and fearful (*M* = 5.65, *SD* = 0.58) faces than for neutral (*M* = 5.09, *SD* = 0.32) faces (*p*s < 0.005), with no differences between happy and fearful faces (*p* > 0.9).

Each stimulus screen was displayed for 200 ms, following by an inter-stimulus interval of 450–650 ms. Experimental task consisted of two standard-deviant conditions (i.e., a happy-deviant-neutral-standard condition and a fearful-deviant-neutral-standard condition) and two reverse-standard-deviant conditions (i.e., a happy-standard-neutral-deviant condition and a fearful-standard-neutral-deviant condition). The presentation orders of the experimental conditions were counterbalanced across participants. Each condition included three blocks with a rest provided between blocks. In each block, ten standards were presented at the very beginning to establish sensory memory trace, and 30 deviants (*P* = 0.18) were then delivered among 138 standards (*P* = 0.82) in a pseudorandom way such that no less than two standards were delivered between consecutive deviants. Participants were asked to ignore the facial stimuli and to detect unpredictable changes in size of a fixation cross (0.80 × 0.80°) appearing in the center of the screen. The fixation cross became either larger (1.03 × 1.03°, 8 times) or smaller (0.57 × 0.57°, 8 times) from time to time, which never occurred simultaneously with facial stimuli. Participants were instructed to press one button when the fixation cross became larger and the other when it became smaller, with their left or right index finger as accurately and rapidly as possible. Buttons were reversed for half of the participants. This primary detection task was run to prevent participants from attending to facial stimuli. Several practice trials were provided prior to the experimental task for familiarization.

### Recording and Analysis

The EEG was recorded at 30 scalp locations using Ag/AgCl electrodes according to the extended 10–20 system (FP1, FP2, F7, F3, Fz, F4, F8, FT7, FC3, FCz, FC4, FT8, T7, C3, Cz, C4, T8, TP7, CP3, CPz, CP4, TP8, P7, P3, Pz, P4, P8, O1, Oz, O2). The EEG signals were referenced to the tip of the nose. The horizontal EOG was recorded via a pair of electrodes placed at the external canthi of each eye to monitor horizontal eye movements. The vertical EOG was recorded via a pair of electrodes placed above and below the left eye to detect vertical eye movements and blinks. The EEG and EOG were amplified and digitalized via a Neuroscan NuAmps amplifier with a band-pass of 0.1–100 Hz and a sampling rate of 500 Hz. Electrode impedance was kept under 5 KΩ throughout the experiment.

The EEG data were analyzed using EEGLAB toolbox (Delorme and Makeig, 2004) and in-house codes under MATLAB environment (MathWorks, Natick, MA, USA). The EEG was filtered with a low-pass of 30 Hz (roll-off 6 dB/octave) and then was segmented into epochs from 100 ms pre-stimulus to 600 ms post-stimulus with the pre-stimulus activity serving as the baseline. The epoched data were screened manually for artifacts (e.g., spikes, drifts and non-biological signals) and then were entered into an informax independent component analysis (runica; Jung et al., 2001; Delorme and Makeig, 2004). Individual components were inspected and blink components were removed. The blink components in all datasets had a large EOG contribution and a frontal scalp distribution. To remove additional artifacts, a semiautomated procedure (Foti et al., 2011) was applied with artifacts defined as follows: a step more than 50 μV between sample points, a voltage difference exceeding 200 μV within a trial, or a maximum voltage difference less than 0.5 μV within 100-ms intervals. Moreover, we utilized an algorithm to remove the trials during which participants blinked while the facial stimuli were still on the screen and thus failed to process the stimuli (Lopez-Calderon and Luck, 2014). Finally, the cleaned data were averaged across trials for each condition and for each participant. Preliminary analysis on the number of the accepted ERP trials revealed no significant effects associated with sex (*p*s > 0.05) for both the happy-neutral context (happy deviants: 78 ± 6 for females and 85 ± 5 for males; happy standards: 277 ± 16 for females and 288 ± 18 for males; neutral deviants: 82 ± 5 for females and 81 ± 9 for males; neutral standards: 280 ± 14 for females and 273 ± 33 for males) and the fearful-neutral context (fearful deviants: 80 ± 8 for females and 79 ± 7 for males; fearful standards: 273 ± 20 for females and 269 ± 26 for males; neutral deviants: 82 ± 6 females and 79 ± 14 males; neutral standards: 280 ± 15 for females and 270 ± 50 for males).

Three ERP components were scored using the local-peak approach (i.e., searching for the largest point that is surrounded on both sides by smaller points) in different time windows over the occipito-temporal regions (i.e., P7 and P8): the P1 (60–130 ms), the N170 (100–200 ms), and the P2 (200–300 ms). vMMNs were created by subtracting the ERPs to standards from those to deviants, separately for the four experimental conditions. Due to the reverse manipulations, the subtractions were performed for the physically identical stimuli and thus resulted in four types of vMMNs (a happy vMMN: happy deviants minus happy standards, a neutral vMMN in the happy context: neutral deviants minus neutral standards, a fearful vMMN: fearful deviants minus fearful standards, and a neutral vMMN in the fearful context: neutral deviants minus neutral standards). Based on previous vMMN literature and the visual inspection of current waveforms, two subcomponents for each type of vMMN were scored as the mean amplitude of two time windows over the occipito-temporal (P7 and P8) and fronto-central (FCz and Cz) regions: the early vMMN (100–200 ms) and the late vMMN (250–350 ms).

Repeated measures ANOVAs were used for all statistical tests and were performed for the happy-neutral context and the fearful-neutral context, respectively. Peak amplitudes of each ERP component were analyzed using sex (male vs. female) as a between-subjects factor and type (deviant vs. standard), emotion (happy vs. neutral for the happy-neutral context; fearful vs. neutral for the fearful-neutral context), and hemisphere (left vs. right) as within-subjects factors. Peak latency results were not reported as they were less theoretically relevant to the present study. Mean amplitudes of each vMMN at occipito-temporal sites were analyzed with a Sex × Emotion × Hemisphere ANOVA. Mean amplitudes of each vMMN at fronto-central sites were analyzed with a Sex × Emotion × Site (FCz vs. Cz) ANOVA. Greenhouse-Geisser epsilon (G-GE) correction was applied for the violation of sphericity when necessary and Bonferroni correction was used for *post hoc* comparisons.

## Results

### Behavioral Performance

Reaction times and accuracy rates for the detection of occasional changes of the fixation cross were compared between female and male groups using an independent-sample *t*-test. Both groups exhibited high accuracy rates for the change detection of the fixation cross (females: *M* = 99.43%, *SD* = 0.64, males: *M* = 99.51%, *SD* = 0.72), *t*_(36)_ = −0.36, *p* = 0.742. Although average reaction times were longer for female group (*M* = 476.65 ms, *SD* = 56.93) compared to male group (*M* = 440.99 ms, *SD* = 64.22), it failed to reach significance, *t*_(36)_ = 1.81, *p* = 0.078.

### Electrophysiological Data

#### P1, N170 and P2 Components

**Figure 2:**
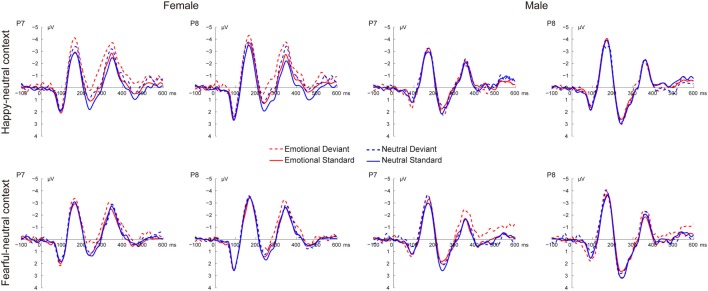
Grand average event-related potential (ERP) waveforms elicited by deviant and standard facial expressions at P7 and P8 for females and males in the happy-neutral and the fearful-neutral contexts.

Figure 2 shows the grand average ERP waveforms at occipito-temporal sites (P7 and P8) elicited by standard and deviant stimuli for both females and males, respectively. As shown in Figure 2, all facial stimuli elicited the canonical P1, N170 and P2 components even when presented outside the focus of attention.

##### The P1

For the happy-neutral context, there was a significant main effect of sex, *F*_(1,36)_ = 4.58, *p* = 0.039, $\eta_{\text{p}}^{2}$ = 0.11, with a larger P1 for females than for males. Moreover, the P1 was enhanced over the right relative to the left hemisphere, as revealed by a significant main effect of hemisphere, *F*_(1,36)_ = 7.90, *p* = 0.008, $\eta_{\text{p}}^{2}$ = 0.18. For the fearful-neutral context, fearful faces elicited an increased P1 relative to neutral faces, *F*_(1,36)_ = 4.20, *p* = 0.048, $\eta_{\text{p}}^{2}$ = 0.10. Similarly, the main effect of sex was significant, *F*_(1,36)_ = 6.24, *p* = 0.017, $\eta_{\text{p}}^{2}$ = 0.15, due to a larger P1 for females than for males. Moreover, the P1 was larger over the right vs. the left hemisphere, *F*_(1,36)_ = 7.70, *p* = 0.009, $\eta_{\text{p}}^{2}$ = 0.18.

##### The N170

During the happy-neutral context, deviants elicited a larger N170 compared to standards, as revealed by a significant main effect of type, *F*_(1,36)_ = 26.03, *p* < 0.0001, $\eta_{\text{p}}^{2}$ = 0.42. This type effect was qualified by a significant two-way interaction between type and sex, *F*_(1,36)_ = 8.29, *p* = 0.007, $\eta_{\text{p}}^{2}$ = 0.19, mainly due to a larger N170 for deviants vs. standards among females (*p* < 0.0001) but not males (*p* = 0.125). With regard to the fearful-neutral context, only a significant main effect of type was obtained, *F*_(1,36)_ = 12.58, *p* = 0.001, $\eta_{\text{p}}^{2}$ = 0.26, with a larger N170 for deviants compared to standards.

##### The P2

For the happy-neutral context, happy relative to neutral faces elicited a less positive P2, *F*_(1,36)_ = 5.73, *p* = 0.022, $\eta_{\text{p}}^{2}$ = 0.14. Moreover, the P2 was less positive over the left compared to the right hemisphere, *F*_(1,36)_ = 10.54, *p* = 0.003, $\eta_{\text{p}}^{2}$ = 0.23. Importantly, there was a significant two-way interaction between type and sex, *F*_(1,36)_ = 4.87, *p* = 0.034, $\eta_{\text{p}}^{2}$ = 0.12. *Post hoc* comparisons revealed that deviants elicited a less positive P2 compared to standards among females (*p* = 0.022) but not males (*p* = 0.477). For the fearful-neutral context, the P2 was less positive over the left hemisphere than over the right hemisphere, *F*_(1,36)_ = 7.65, *p* = 0.009, $\eta_{\text{p}}^{2}$ = 0.18. This hemisphere effect seemed to be more pronounced for fearful faces (*p* = 0.003) relative to neutral faces (*p* = 0.044), resulting in a significant two-way interaction between emotion and hemisphere, *F*_(1,36)_ = 5.95, *p* = 0.020, $\eta_{\text{p}}^{2}$ = 0.14.

#### vMMN Components

**Figure 3:**
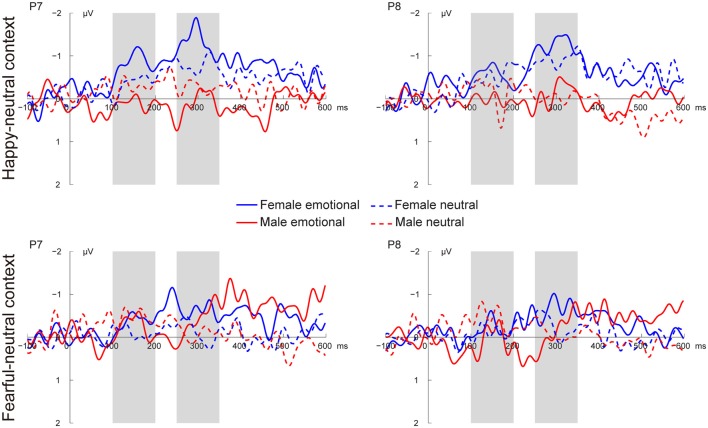
Grand average visual mismatch negativity (vMMN) at occipito-temporal sites (P7 and P8) elicited by emotional and neutral facial expressions for females and males in the happy-neutral and the fearful-neutral contexts.

**Figure 4:**
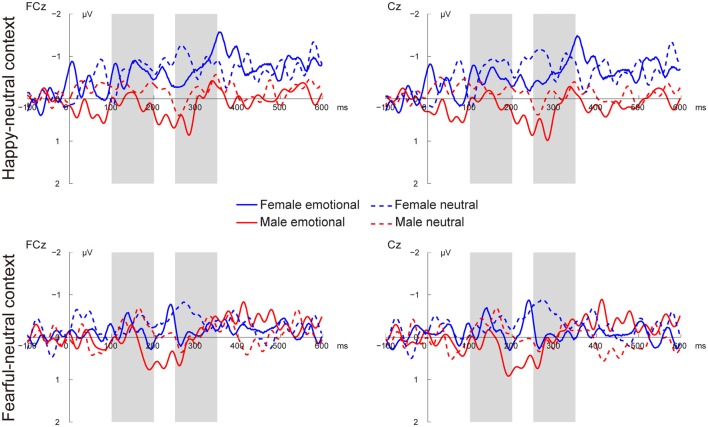
Grand average vMMN at fronto-central sites (FCz and Cz) elicited by emotional and neutral facial expressions for females and males in the happy-neutral and the fearful-neutral contexts.

**Figure 5:**
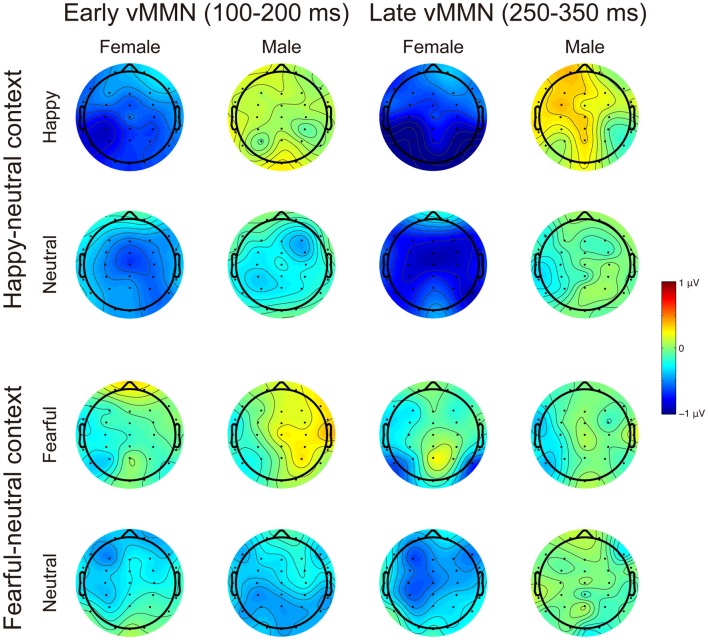
Scalp topographic maps for the early (100–200 ms) and late (250–350 ms) vMMNs in response to emotional and neutral facial expressions for females and males in the happy-neutral and the fearful-neutral contexts.

Figures 3, 4 present the grand average vMMNs, calculated as deviants minus standards, elicited by the physically identical stimuli in the happy-neutral and fear-neutral contexts for both females and males. The topographic maps for the vMMNs are displayed in Figure 5, showing an occipito-temporal distribution and a fronto-central distribution in two different intervals (100–200 ms and 250–350 ms), which are consistent with previous research.

##### The early vMMN

For the happy-neutral context, females exhibited a larger vMMN than males over both the occipito-temporal regions, *F*_(1,36)_ = 4.45, *p* = 0.042, $\eta_{\text{p}}^{2}$ = 0.11, and the frontocentral regions, *F*_(1,36)_ = 4.18, *p* = 0.048, $\eta_{\text{p}}^{2}$ = 0.10. For the fearful-neutral context, no significant effects were found (*p*s > 0.05).

##### The late vMMN

**Figure 6:**
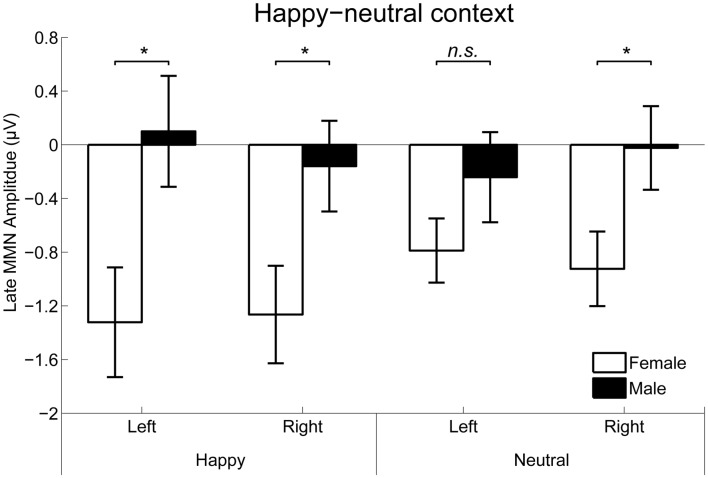
The Sex × Emotion × Hemisphere interaction for the mean amplitude of the late vMMN over the occipito-temporal areas. Error bars represent standard error. *p < 0.05, n.s., non-significant.

For the happy-neutral context, the main effect of sex was significant over both the occipito-temporal regions, *F*_(1,36)_ = 7.59, *p* = 0.009, $\eta_{\text{p}}^{2}$ = 0.17, and the frontal-central regions, *F*_(1,36)_ = 5.94, *p* = 0.020, $\eta_{\text{p}}^{2}$ = 0.14, with an enhanced vMMN for females compared to males. Critically, there was a significant three-way interaction of Sex × Emotion × Hemisphere over the occipito-temporal regions (Figure 6), *F*_(1,36)_ = 4.34, *p* = 0.044, $\eta_{\text{p}}^{2}$ = 0.11. *Post hoc* comparisons revealed that females showed more negative vMMN amplitudes compared to males over both the left (*p* = 0.019) and the right (*p* = 0.032) hemispheres for happy faces. For neutral faces, however, the sex effect was present over the right hemisphere (*p* = 0.038), but not the left hemisphere (*p* = 0.194). During the fearful-neutral context, no significant effects were found (*p*s > 0.2).

## Discussion

Using a reverse-standard-deviant paradigm, the present study compared the vMMN, an index of automatic change detection, in response to unattended facial expressions between females and males. Both females and males showed comparable accuracy rates and reaction times for the detection of the unpredictable changes of the size of a fixation cross, indicating that the two groups did not differ in their overall task engagement. We found a larger P1 for females compared to males for all facial expressions. Moreover, females relative to males were more sensitive to the differences between deviants and standards in the happy-neutral context, as revealed by a more negative N170 and a less positive P2 for deviants vs. standards in females but not males. This greater sensitivity in females was further supported by vMMN findings. During the early stage (100–200 ms), females displayed more negative vMMN responses to both happy and neutral faces than males over the occipito-temporal and fronto-central regions. During the late stage (250–350 ms), females relative to males exhibited more negative vMMN responses to both happy and neutral faces over the fronto-central and right occipito-temporal regions, but only more negative vMMN responses to happy faces over the left occipito-temporal region. In contrast, no sex differences were found for vMMN responses in the fearful-neutral context.

A number of studies have demonstrated that females are more emotionally perceptive than females (Whittle et al., 2011). In the current study, we found a larger P1 for females than males for both emotional (happy and fearful) and neutral faces appearing in the visual periphery when their attention was engaged in a visual detection task in the center of the visual field. These findings are consistent with previous studies reporting a larger P1 for females vs. males (Lee et al., 2010) but extend these studies to show that sex differences could occur during non-attentional conditions. Furthermore, we found a more negative N170 and a less positive P2 for deviant facial expressions than for standard facial expressions in females but not males, which are in line with previous research (Xu et al., 2013). Moreover, the sex differences in both the N170 and P2 time windows were observed in the happy-neutral context, but not the fearful-neutral context, indicating a female advantage for processing positive emotions during non-attentional conditions.

The vMMN is thought to reflect the automatic detection of mismatches between a sensory input and the predictive memory representation generated by repeated standard stimuli (Czigler, 2007). Whereas most previous studies focused on sex differences in facial expressions on the conscious level (Whittle et al., 2011), the unconscious processing of facial expressions between females and males has been largely ignored. Given that sex differences have been reported in the early stage of facial expression processing (Campanella et al., 2004; Lee et al., 2010), it is possible that sex differences in facial expressions can occur during the pre-attentive stage. Using schematic faces, a previous study reported a larger vMMN (120–230 ms) in response to sad vs. happy faces over the right hemisphere for females, but not for males (Xu et al., 2013). In line with this study, we found that the pre-attentive processing of facial expressions was modulated by sex during the similar time window (100–200 ms). Specifically, females exhibited a greater level of vMMN responses compared to males in the occipito-temporal regions during the early processing stage, which appeared for both happy and neutral faces during the happy-neutral context. These findings suggest that females relative to males are more sensitive to the changes of both happy and neutral facial expressions during this stage. This early vMMN finding corresponded with the latency (peaking around 165 ms) and scalp topography (the occipito-temporal region) of the well-known face-sensitive N170 component, wherein females relative to males displayed larger N170 amplitudes for deviant vs. standard facial expressions during the happy-neutral context. The N170 reflects the structure encoding of faces (Bentin et al., 1996) as well as is sensitive to emotional expressions (Eimer and Holmes, 2007). It is thus possible that the early vMMN represents the visual processing of deviant and standard stimuli reflected by the N170, but these two processes cannot be differentiated in the current study.

Whereas the early vMMN findings revealed a female advantage in the automatic change detection of facial expressions, the late (250–350 ms) vMMN findings were more supportive of a female advantage in the automatic change detection of happy facial expression, rather than general facial expressions. Specifically, females compared to males exhibited a larger vMMN for both happy and neutral expressions over the fronto-central and right occipito-temporal regions, as the early vMMN did. Over the left hemisphere, however, females relative to males showed an enhanced late vMMN for happy facial expressions, with no sex differences for neutral facial expressions. Our late vMMN findings suggest that the left occipito-temporal region plays an important role in the female advantage in the automatic change detection of happy facial expressions. Supporting this idea, a well-known theory of emotional processing, the valence hypothesis, proposes that the left hemisphere is dominant for positive emotion processing whereas the right hemisphere is specialized in processing negative emotion processing (Davidson et al., 1990; Prete et al., 2015b). Indeed, using happy and fearful faces in a reverse-standard-deviant design, a previous study reported more negative vMMN responses to happy vs. fearful facial expressions over left temporal areas (Stefanics et al., 2012).

Surprisingly, we failed to find any sex differences in vMMN responses during the fearful-neutral context. There are several possible explanations about this finding. In order to elicit the vMMN, a predictive memory representation should be established by repeated standard stimuli and a violation of the predictive representation should occur. It is thus possible that the predictive memory representation failed to be generated in the fearful-neutral context. In our task, each stimulus screen consisted of two female faces and two male faces with the same expression and were shuffled randomly around four locations in the visual periphery. To generate a prediction error signal, the visual system has to extract the common feature across the four faces, that is, the emotional valence, and then establish a predictive memory representation. On the one hand, fearful faces might be more different from each other than happy faces and thus it was more difficult for participants to extract the common fearful facial expression. In consistent with this explanation, a recent theory has proposed that negative information is less similar than positive information (Alves et al., 2017). In this regards, the fearful faces might consist of more heterogeneous exemplars than the happy faces in the current study and thus were more difficult to be integrated. On the other hand, females relative to males might be more capable to discriminate fearful faces, as demonstrated in previous research (Whittle et al., 2011), such that these fearful faces were categorized on a more subtle level than happy faces in females. This appears to make it more difficult to generate a predictive memory representation in females compared to males, resulting in no sex differences observed during the fearful-neutral context. Both possibilities could be responsible for no sex differences in the fearful-neutral context. Unfortunately, they cannot be discriminated in the current study, which warrants further studies. A third possibility is linked to the possible subcortical involvement for fearful facial expressions. Specifically, convergent evidence highlights a subcortical face-detection pathway involving the superior colliculus, pulvinar and amygdala, which is especially sensitive to fearful facial expressions (for a review, see Johnson, 2005). Unfortunately, neural generators from the subcortical route are difficult, if not possible, to be detected by the scalp-recorded EEG (Luck, 2014).

## Conclusion

The current study investigated sex differences in the automatic detection of changes in facial expressions during a happy-neutral context and a fearful-neutral context. Females relative to males demonstrated stronger automatic processes of general facial expressions in early processing stage. During the late stage, despite a female advantage for general facial expressions over the fronto-central and right occipito-temporal regions, females exhibited a greater sensitivity to detecting pre-attentively the changes of happy facial expressions over the left occipito-temporal region than males. In addition, these sex differences were limited to the happy-neutral context, instead of the fearful-neutral context. Together, our findings revealed dynamic differences in the automatic neural processing of facial expressions between females and males in the happy-neutral context.

## Author Contributions

YZ, QL and XL: conceived and designed the study. YX and SZ: data collection and analysis. QL and YZ: wrote the article.

## Conflict of Interest Statement

The authors declare that the research was conducted in the absence of any commercial or financial relationships that could be construed as a potential conflict of interest.
